# Nanomedicine for lymphangioleiomyomatosis: toward targeted and durable anti-fibrotic therapy

**DOI:** 10.1097/MS9.0000000000003889

**Published:** 2025-09-16

**Authors:** Ashish Ashok Lal, Muneeb Faiz, Maliha Khalid, Muhammad Talha, Aminath Waafira

**Affiliations:** a Department of Medicine, Jinnah Sindh Medical University, Karachi, Pakistan; b Department of Medicine, King Edward Medical University, Lahore, Pakistan; c School of Medicine, The Maldives National University, Malé, Maldives

**Keywords:** anti-fibrotic therapy, lipid nanoparticles, lymphangioleiomyomatosis, nanomedicine

## Abstract

Lymphangioleiomyomatosis (LAM) is a rare cystic lung disease primarily affecting women, driven by TSC1 or TSC2 mutations that lead to constitutive mTORC1 activation and progressive respiratory failure. While rapamycin remains the only approved treatment, it is disease-stabilizing rather than disease-reversing. Recent studies demonstrate that lung-targeted lipid nanoparticles delivering functional TSC2 mRNA can restore tumor suppressor activity, inhibit mTORC1 signaling, and reduce cystic lung damage in preclinical models, offering a potential disease-modifying approach. Effective nanomedicine delivery to LAM lungs faces significant barriers, including mucus entrapment, altered airflow, and fibrotic remodeling, necessitating mucus-penetrating formulations for uniform deposition. Advances in nanoparticle-based anti-fibrotic therapy from related conditions such as liver fibrosis support the translational promise of this approach. Clinical trials are urgently needed to optimize design, evaluate safety, and assess efficacy in patients with LAM, with the goal of achieving targeted, durable, and personalized therapy.

*Dear Editor,*

Lymphangioleiomyomatosis (LAM) is a rare lung disease affecting women of reproductive age, caused by abnormal growth of smooth muscle-like cells that cause cystic destruction of lung tissue and cause respiratory failure. Most cases involve TSC1 or TSC2 mutations, leading to constant mTORC1 activation and uncontrolled cell growth. Rapamycin, the only FDA-approved therapy, slows progression but cannot reverse damage. Recent studies highlight lung-targeted lipid nanoparticles (LNPs) delivering TSC2 mRNA as a promising strategy to restore normal signaling and reduce cystic destruction^[1]^.

Lung-targeted LNPs delivering functional Tsc2 mRNA successfully restored TSC2 activity in TSC2-null cells, suppressing mTORC1 signaling and reducing phospho-S6 expression in both *in vitro* TSC2-deficient cell lines and *in vivo* mouse models. Tsc2 mRNA nanoparticles significantly decreased tumor burden, reduced cellular proliferation, and repressed mTORC1 activity. These findings provide strong proof of concept that restoring tumor suppressor genes via organ-specific mRNA delivery through nanoparticles may offer a powerful, disease-modifying strategy in the treatment of LAM, in contrast to rapamycin, which only inhibits downstream signaling. By restoring wild-type TSC2, it reestablishes normal cellular regulation and offers the potential for a more durable, disease-modifying treatment^[2]^.

Pulmonary delivery of nanomedicine in LAM is hindered by the lung’s defense systems. Inhaled particles are rapidly cleared via mucociliary transport in the upper airways or engulfed by alveolar macrophages if 1–3 μm in size, while those <200 nm often escape immune clearance but are exhaled before deposition. Moreover, the cystic and fibrotic remodeling seen in LAM disrupts airflow and particle distribution, making targeted delivery to diseased regions even more difficult^[3]^. The airway mucus layer poses a key barrier by trapping adhesive nanoparticles and preventing their penetration into deeper lung zones. Mucus-penetrating formulations are essential for uniform and sustained distribution, enabling access to cystic and fibrotic regions. In LAM, altered mucus and surfactant properties intensify this obstacle; achieving efficient nanodelivery requires overcoming mucus entrapment to ensure adequate retention in diseased lungs^[4]^.

Recent advancements have proven that nanocarriers such as liposomes, polymeric nanoparticles, and inorganic nanostructures can encapsulate anti-fibrotic drugs and deliver them effectively to fibrotic lesions, thereby improving therapeutic outcomes in fibrotic diseases like liver fibrosis that share mechanistic aspects with LAM. These nanoformulations diffuse and stabilize drugs and prevent the off-target toxicity of the drugs by confining the therapeutic effects of their action to the site of disease. Moreover, theranostic nanoplatforms incorporating diagnostic and therapeutic functions can be used to monitor treatment response in real time, which allows therapeutic modification to be personalized. Although clinical trials directly evaluating nanomedicine for LAM are lacking, substantial success with related fibrotic diseases supports their possible translation to LAM management^[5,6]^. Our work is in line with the TITAN Guidelines on the need for transparency in artificial intelligence use in health care^[7]^.

To summarize, nanomedicine appears to be a game-changing approach to overcoming current challenges in therapeutic techniques for LAM in targeted, effective, and safer anti-fibrotic treatment. Insistence on research and clinical trials is required for further optimization of nanocarrier design, studying long-term safety, and validating efficacy in patients with LAM. The incorporation of nanomedicine in LAM would improve patient outcomes and quality of life, even as it demands funding and interdisciplinary cooperation in this area.

## Data Availability

No data were generated for this manuscript.

## Research registration unique identifying number (UIN)

Name of the registry: N/A Unique Identifying number or registration ID: N/A Hyperlink to your specific registration (must be publicly accessible and will be checked): N/A
